# Supplementary material for: Recovery of logged forest fragments in a human-modified tropical landscape during the 2015-16 El Niño
Source: Nat Commun. 2021 Mar 9;12:1526. doi: 10.1038/s41467-020-20811-y (PMC7943823; doi:10.1038/s41467-020-20811-y)
Supplement: Supplementary file 1 — Supplementary Information [file 41467_2020_20811_MOESM1_ESM.docx]

**Recovery of logged forest fragments in a human-modified tropical landscape during the 2015-16 El Niño**

Matheus Henrique Nunes^1,2^*, Tommaso Jucker^1,3^, Terhi Riutta^4,5^, Martin Svátek^6^, Jakub Kvasnica^6^, Martin Rejžek^6^, Radim Matula^7^, Noreen Majalap^8^, Robert M. Ewers^4^, Tom Swinfield^1^, Rubén Valbuena^1,9^, Nicholas R. Vaughn^10^, Gregory P. Asner^10^ and David A. Coomes^1^*

^1^ Department of Plant Sciences and the Conservation Research Institute, University of Cambridge, Cambridge CB2 3QZ, UK.

^2^ Department of Geosciences and Geography, University of Helsinki, Helsinki, 00014, Finland

^3^ School of Biological Sciences, University of Bristol, Bristol, BS8 1TH, UK

^4^ Department of Life Sciences, Imperial College London, Silwood Park Campus, Buckhurst Road, Ascot, Berkshire, SL5 7PY, UK

^5^ School of Geography and the Environment, Environmental Change Institute, University of Oxford, Oxford, OX1 3QY, United Kingdom

^6^ Department of Forest Botany, Dendrology and Geobiocoenology, Faculty of Forestry and Wood Technology, Mendel University in Brno, 613 00, Czech Republic

^7^ Department of Forest Ecology, Faculty of Forestry and Wood Sciences, Czech University of Life Sciences Prague, Kamýcká 129, Prague, 165 00, Czech Republic

^8^ Sabah Forestry Department, Sandakan, 90009, Malaysia

^9^ School of Natural Sciences, Bangor University, Gwynedd, LL57 2UW, UK

^10^ Center for Global Discovery and Conservation Science, Arizona State University, Tempe AZ and Hilo HI USA

* Corresponding authors.

**SUPPLEMENTARY INFORMATION**

**Supplementary Methods 1. LiDAR data fusion and canopy height change estimation**

The first LiDAR data were acquired in November 2014 using a Leica ALS50‐II LiDAR sensor flown by NERC's Airborne Research Facility. The sensor emitted pulses at a frequency of 120 kHz, had a field of view of 12° and a footprint of about 40 cm, with a mean point (i.e. return) density of about 13.2 points m^-2^ (+13.2 points m^-2^ standard deviation). The second LiDAR survey was conducted by the ASU Global Airborne Observatory (GAO; formerly the Carnegie Airborne Observatory[^1^](https://paperpile.com/c/rfZfnr/wtQ9u)) in April 2016. The GAO ALS system was set to a combined-channel pulse frequency of 200 kHz, a field of view of 34° and a footprint of about 1.8 m, yielding a mean point density of 4.1 points m^-2^ (±2.2 points m^-2^ standard deviation).

LiDAR-based canopy heights were obtained using different sensor configurations and flight parameters, which can affect canopy height estimation [^2^](https://paperpile.com/c/rfZfnr/WU2Cl). To minimise errors in the fusion of both LiDAR datasets, we (i) created a common digital terrain model (DTM) using a combination of ground returns from both LiDAR surveys, (ii) coarsened pixel resolution to reduce uncertainties due to artefacts of repeat LiDAR data such as wind direction and within-canopy variation, (iii) restricted our analysis to areas with high point density in the 2014 NERC LiDAR survey and (iv) tested the sensitivity of our results to spatial variation in point density in the 2016 GAO LiDAR survey.

Firstly, a common digital terrain model (DTM) at 1 m resolution was created using a combination of ground returns from both surveys. Using a TIN-densifying algorithm available in “lasground” tool, LiDAR returns were classified as ground and non-ground, and their heights above ground were calculated by subtracting the elevation of the resulting DTM underneath each of them. Then a canopy height model (CHM) representing the height of vegetation was generated separately for each survey, following the methodology outlined by Khosravipour and colleagues [^3^](https://paperpile.com/c/rfZfnr/Fryr), and top-of-canopy height aboveground (TCH) was initially calculated as the mean CHM at 2 m resolution. It has been demonstrated that TCH, as measured by LiDAR, is a useful metric for estimating structural attributes of natural tropical forests and is relatively insensitive to sensor and flight specifications [^4^](https://paperpile.com/c/rfZfnr/PXen).

Variance of top-of-canopy height (TCH) change can be overestimated owing to artefacts of repeat LiDAR data such as wind direction and within-canopy variation. We used a subset of TCH change data to investigate how bilinear resampling would affect the mean and variance of TCH change. Mean values were not affected by coarsening pixels from 2 m to lower resolutions, however standard deviation (SD) was significantly reduced; SD of 2-m resolution TCH change was 4.9 m, with a significant reduction to 2.1 m for 36-m resolution pixels - when SD was no longer affected by pixel size (Supplementary Fig. 1a, b). The final TCH map was then coarsened from 2 m to 30 m resolution following Asner and colleagues [^5^](https://paperpile.com/c/rfZfnr/PVBQV) given the similar SD values of TCH change to the lower resolution data.


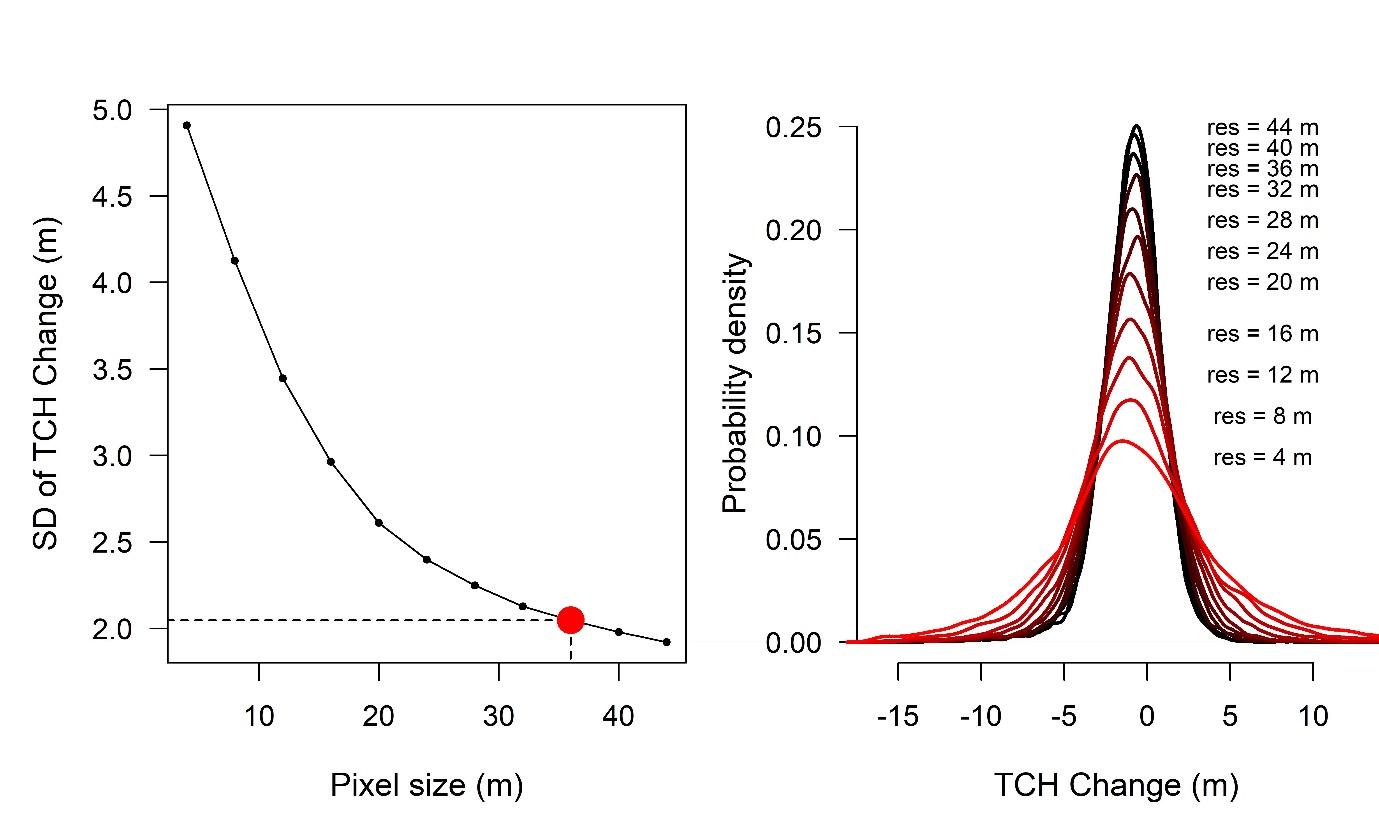


**a**

**b**

**Supplementary Fig. S1: Reducing uncertainties in top-of-canopy height estimates.** Changes in top-of-canopy height (TCH) can be overestimated owing to artefacts of repeat LiDAR data such as wind direction and within-canopy variation. From a subset of the repeat LiDAR data with 5,000 TCH change measurements, **(a)** shows a reduction in standard deviation (SD) and **(b)** the consistent mean values of TCH change with resampling to lower resolution pixels (varying from high resolution in red to low resolution in black).

LiDAR estimates of TCH can be affected by low point density [^6^](https://paperpile.com/c/rfZfnr/3sgz). To test for potential biases arising from differences in point density, we first tested how point density of the 2014 NERC LiDAR survey affected the LiDAR-based TCH change estimates (Supplementary Fig. 2). In comparison with the field-based TCH changes of 0.23 m (95% confidence intervals (CI): -0.30 – 0.77 m) (shaded area), an underestimation of tree height associated with point density < 10 points m^-2^ in the NERC dataset may have contributed to an overestimation of TCH change. Thus, we removed these pixels (~ 62% of the dataset) and only data with point density > 10 points m^-2^ in the NERC dataset were used to avoid canopy height change overestimation.


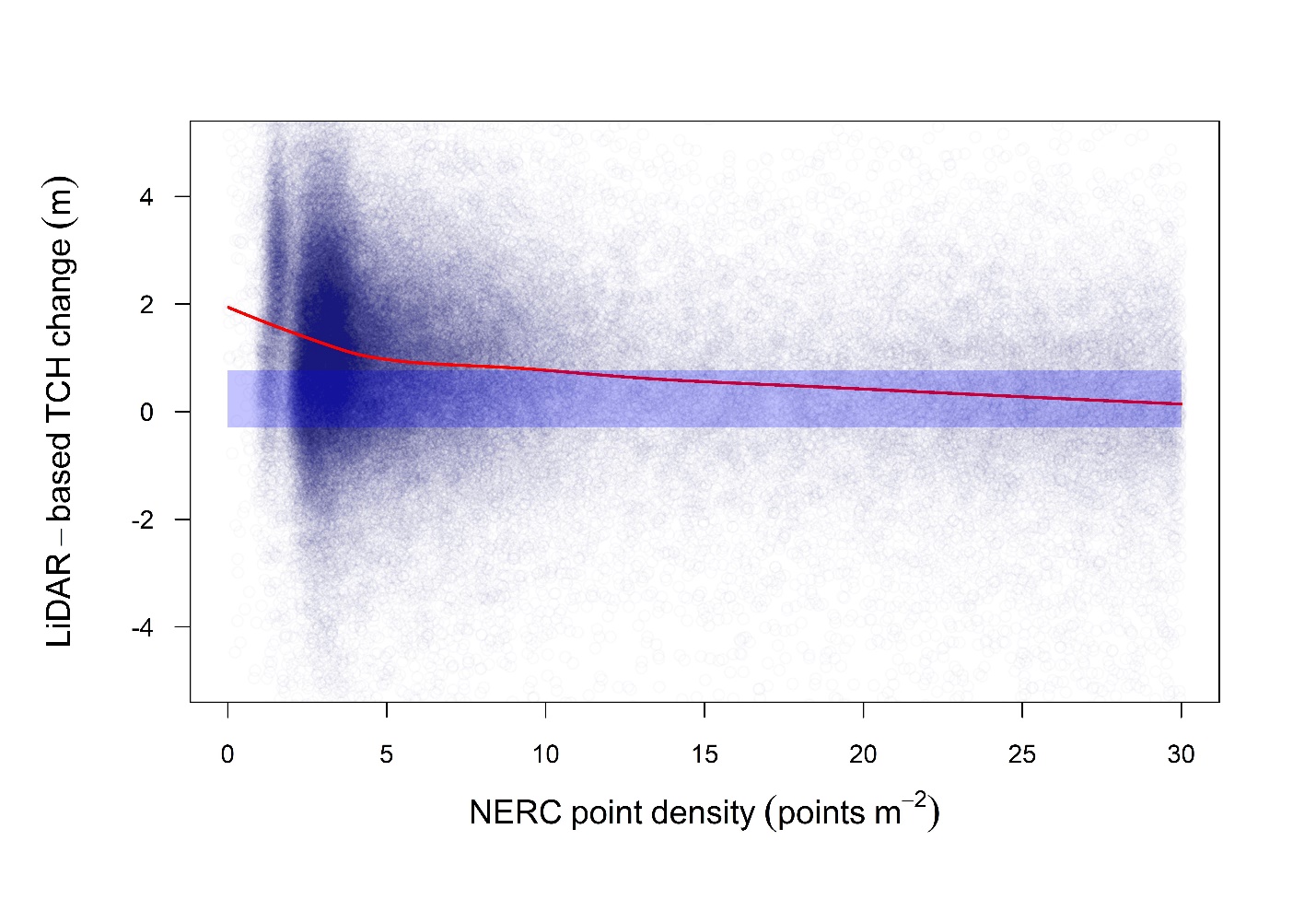
**Supplementary Fig. 2: NERC’s point density effects on top-of-canopy height (TCH) change estimates.** Point density of the 2014 LiDAR from the Natural Environment Research Council **(**NERC) survey versus top-of-canopy height (TCH) change in meters between the two LiDAR surveys. Each point represents a 30 m resolution pixel (n = 96,297 pixels). The red line depicts a smoothing loess function and the shaded blue area represents the field-based TCH change varying between -0.30 – 0.77 m (95% confidence intervals).

We also assessed the influence of point density in the GAO data on TCH change estimation by repeating the same analysis using GAO’s low point density (< 2 points m^-2^) and GAO’s high point density (> 2 points m^-2^) datasets. We demonstrate below that (i) although there are some differences in the spatial distribution of areas with high and low GAO’s point density (Supplementary Fig. 3a, b, c), there is also enough overlap to test for point density effects on tree height change estimations, and (ii) that even if we remove areas with low point density the results are unchanged (Supplementary Fig. 4 versus Fig. 5 of the manuscript), most probably because of the wider beam divergence and higher-wattage lasers. Although low point density areas in the GAO data predominantly cover short forests and high point density data cover tall forests (Supplementary Fig. 3a), our canopy height growth predictions are similar using only high point density data, which is unlikely to happen if point density were affecting these patterns. We did observe less-pronounced edge effects when using the high point density data only (Supplementary Fig. 4 versus Fig. 5 of the manuscript), however this is due to the fact that most forests neighbouring oil palm plantations were covered by low point density data (Supplementary Fig. 3c) and thus we have an insufficient number of pixels to robustly fit the prediction models.

**Supplementary Fig. 3: Spatial distribution of GAO’s point density.** Distribution analysis of areas with low point density ( <2 points m^-2^; red) and high point density ( > 2 points m^-2^; blue) in the data collected by the Global Airborne Observatory (GAO) in relation to **(a)** top-of-canopy height (TCH) in meters, **(b)** topographic position index (TPI) and **(c)** distance from oil palm plantations in meters (n = 36,655 pixels).


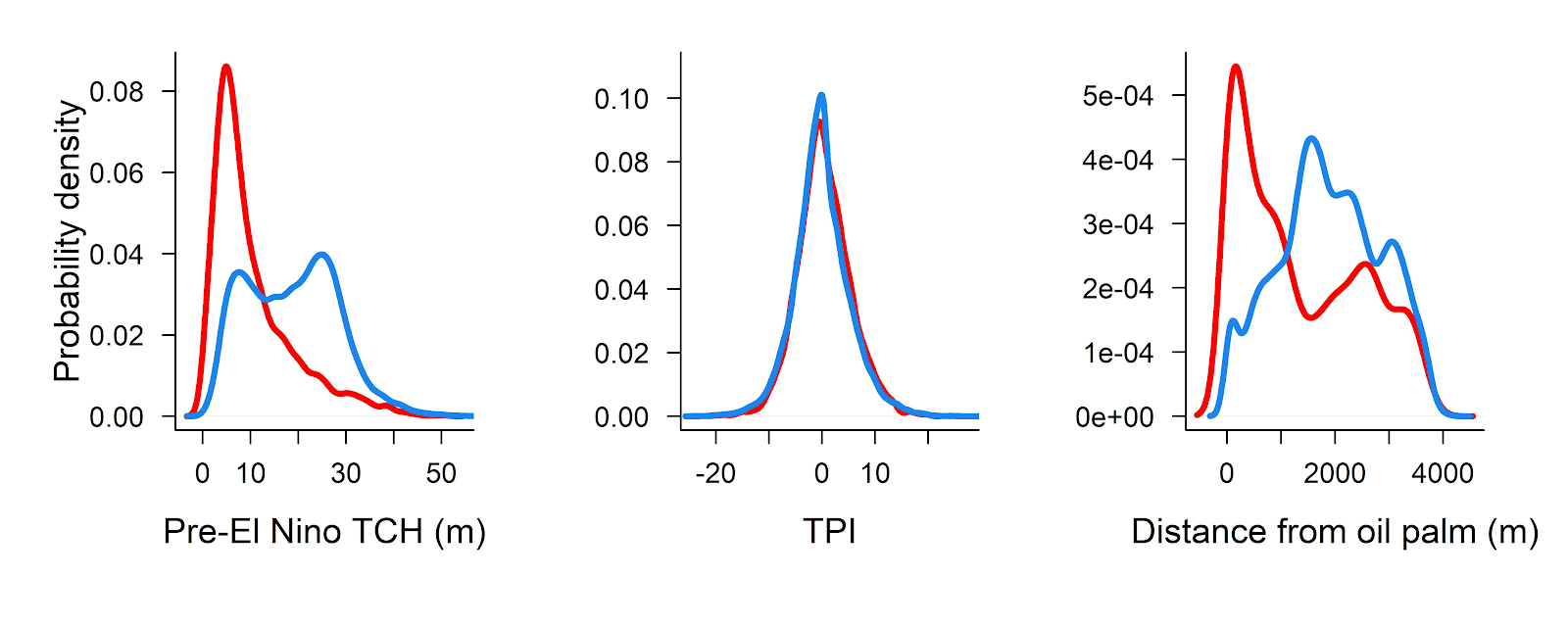


**a**

**c**

**b**


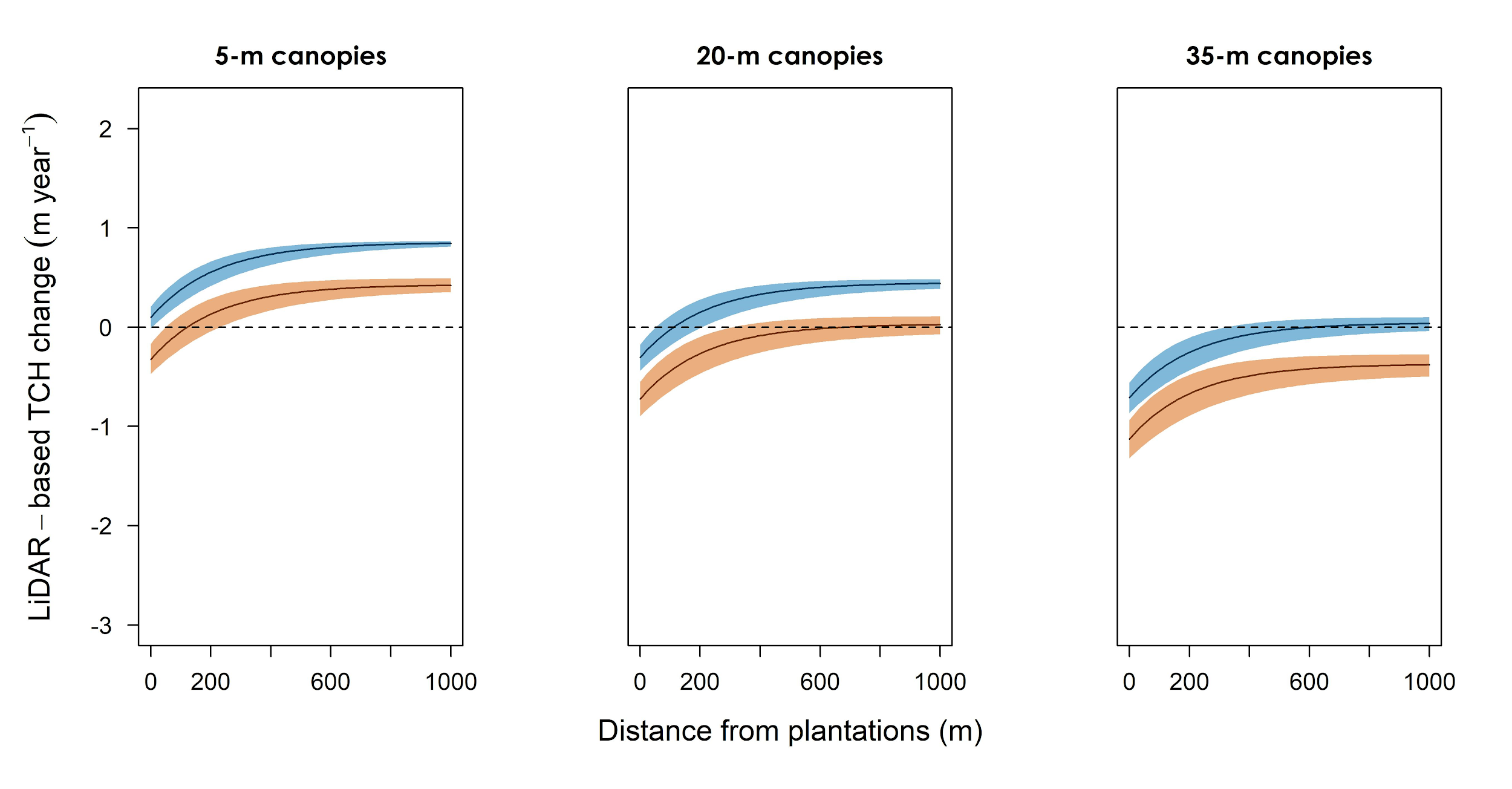
**Supplementary Fig. 4: Canopy height change predictions using GAO’s high point density dataset.** Predicted effects of distance from oil palm plantations and topographic position index (TPI) on top-of-canopy height (TCH) growth per year in forests of different successional stages during the 2015-2016 El Niño in Malaysian Borneo. These predictions were based on a subset of the LiDAR data collected by the Global Airborne Observatory (GAO) with point density above 2 points m^-2^ to investigate whether the predictions were affected by point density in the GAO data. LiDAR-based TCH change per year was predicted by fitting nonlinear models to 25 subsets of the dataset, each containing 5,000 pixels. The black curves are based on median parameter values and CIs are based on their variances (see Supplementary Methods 7**)**. Short, medium and tall canopies were 5 m, 20 m and 35 m in top-of-canopy height (TCH) measured in the first LiDAR survey (November 2014). Valley-bottom (blue) and hilltop (orange) curves correspond with TPI values of -8.2 and 9.0, respectively, from the 5^th^ and 95^th^ quantiles.

.

**Supplementary Methods 2. Spatial distribution of environmental variables**

The repeated high-resolution LiDAR surveys covered 3,300 ha of a forest - oil palm landscape with forest canopy heights varying from 0 to 64 m, with forest edges varying from 0 to nearly 4200 m from oil palm plantations. The undulating landscape had topographic position indices (TPI) varying from -24 (deep valleys) to 35.1 (elevated hilltops) (Supplementary Fig. 5).


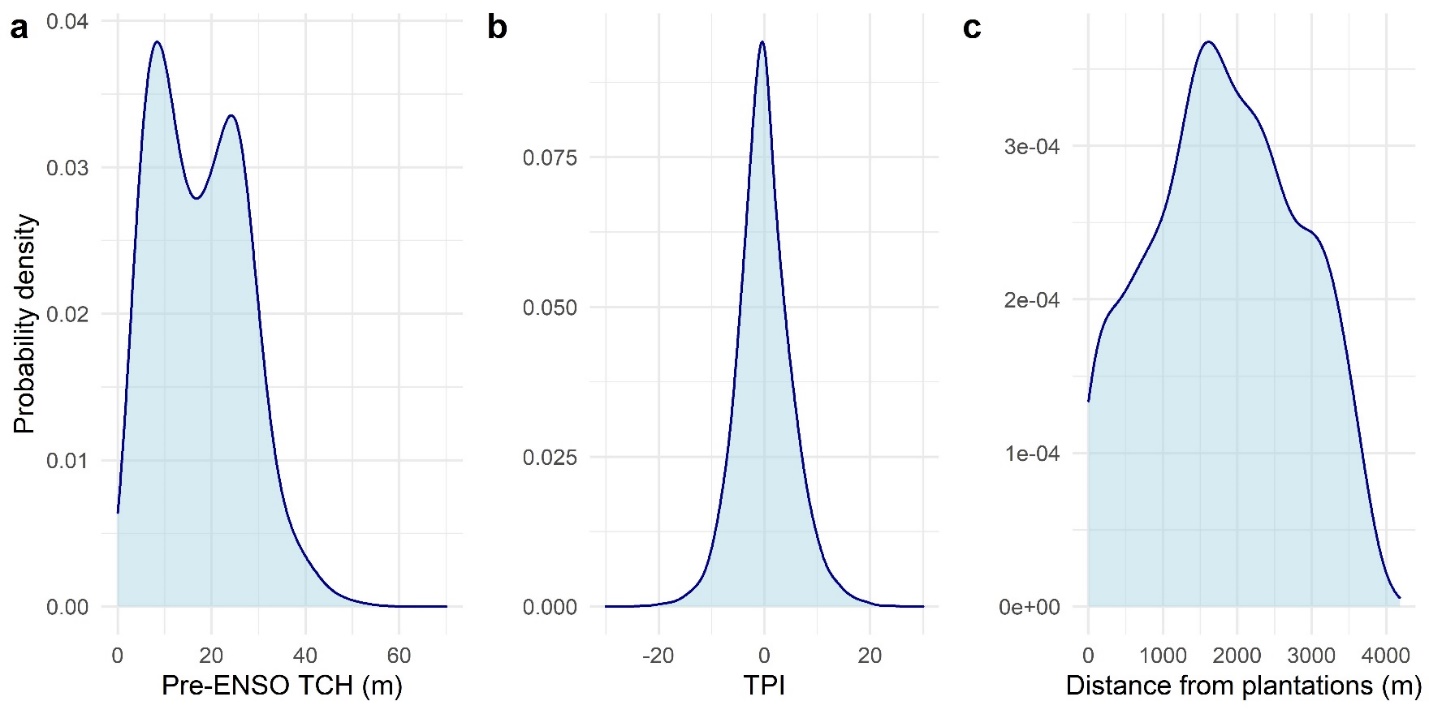


**Supplementary Fig. 5: Spatial distributions of environmental variables.** Spatial distribution of **(a)** the top-of-canopy height (TCH) before the El Niño Southern Oscillation (pre-ENSO) event, **(b)** topographic position index (TPI) and **(c)** distance of forests from oil palm plantations covered by repeated airborne LiDAR data across the SAFE landscape in Malaysian Borneo (n= 36,655 pixels).

**Supplementary Methods 3. Topographic Position Index as a predictor of presence of rivers and water availability**

Based on a stream network of the SAFE Project, we show that low topographic position index (TPI) values are generally associated with rivers (Supplementary Fig. 6, 7a). Negative TPI values are likely to be within ~ 75 m from rivers and 50% of the forests with TPI < -5 are within 15 m from rivers; thereby the low-lying forests of our study generally represent riparian forests.


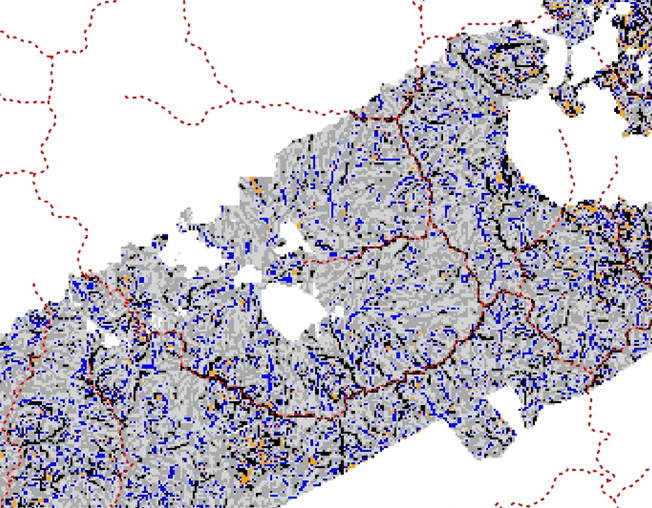


**Supplementary Fig. 6: River network across the SAFE landscape.** A portion of the topographic position index (TPI) map across the Stability of Altered Forest Ecosystems (SAFE) Project landscape. The black pixels represent TPI values < -5. The grey, blue and orange pixels are TPI > -5, with the orange pixels representing the largest TPI values (hilltops). White pixels are NA data due to the presence of clouds. The juxtaposed red dashed lines correspond to rivers from a stream network data for the SAFE Project. The figure demonstrates that areas closer to rivers tend to be areas with low TPI and most of the low-lying areas represent riparian forests.

To further understand how topographic position mediates water availability for trees, we used the DTM to calculate the topographic wetness index (TWI) for each 30 m grid cell using the 'dynatopmodel' R package [^7^](https://paperpile.com/c/rfZfnr/veep). High TWI values indicate grid cells with topographic characteristics favourable for accumulating higher levels of soil moisture, and vice versa [^8^](https://paperpile.com/c/rfZfnr/gjk2). When we compare TPI and TWI values across SAFE, we clearly see that areas with more convex curvature and steeper slopes (higher TPI) tend to have lower TWI values, and vice versa (Supplementary Fig. 7b; Spearman correlation = -0.81, P-value < 0.0001).


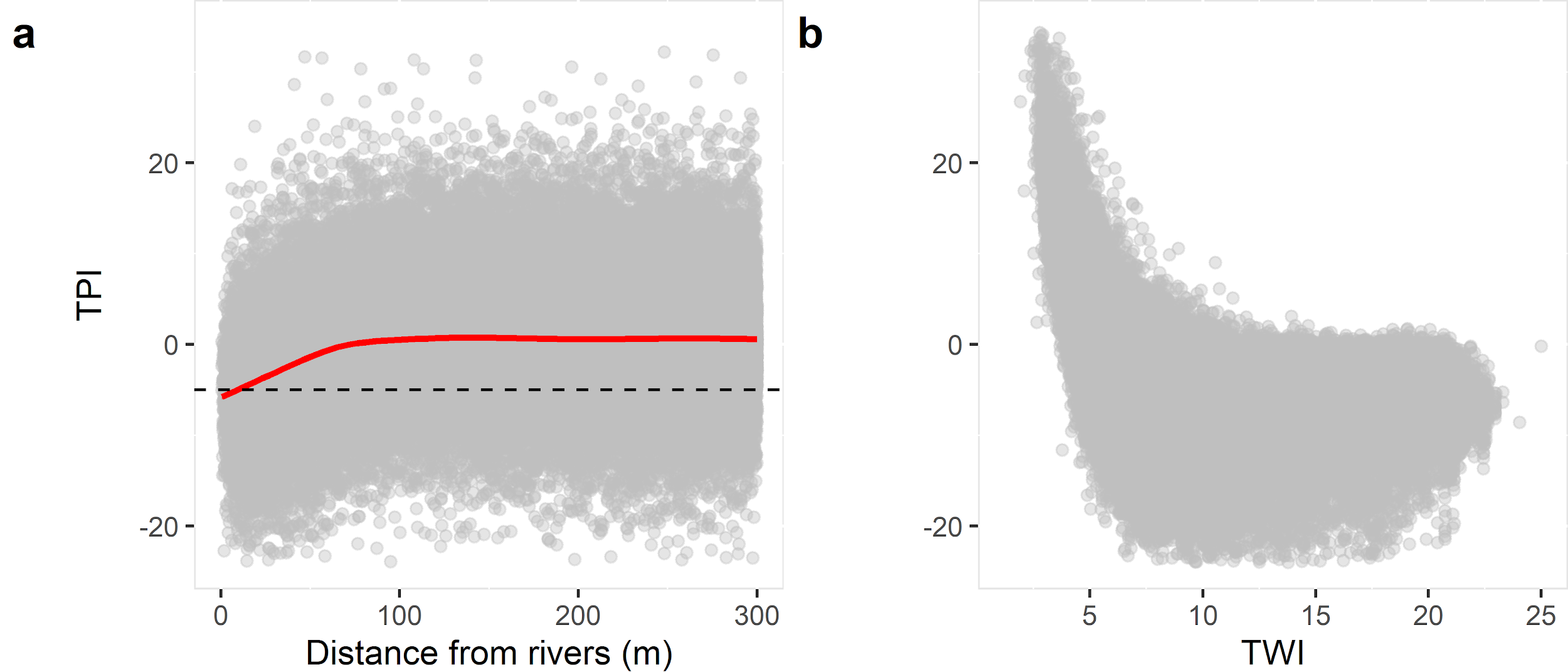


**Supplementary Fig. 7: Relationship of TPI with distance from rivers and water availability.** Topographic position index (TPI) variation with **(a)** distance from rivers in meters and **(b)** topographic wetness index (TWI). The horizontal dashed line represents a TPI value of -5, and the red solid line represents a smoothing loess function indicating that low-lying areas - negative low TPIs - are more likely to be within ~ 75 m from rivers (n = 36,655 pixels).

**Supplementary Methods 4. Field top-of-canopy height calculation**

Tree height (H) and crown area (CA) were locally measured for a total of 3248 trees in the 1-ha Global Ecosystems Monitoring (GEM) permanent plots. We used these data to fit allometric equations S1 and S2 for predicting the H and CA of trees that were not measured (n =10393; Supplementary Fig. 8).

H = 2.21 x DBH ^0.67^  (S1)

CA= 0.22 x DBH ^1.41^  (S2)


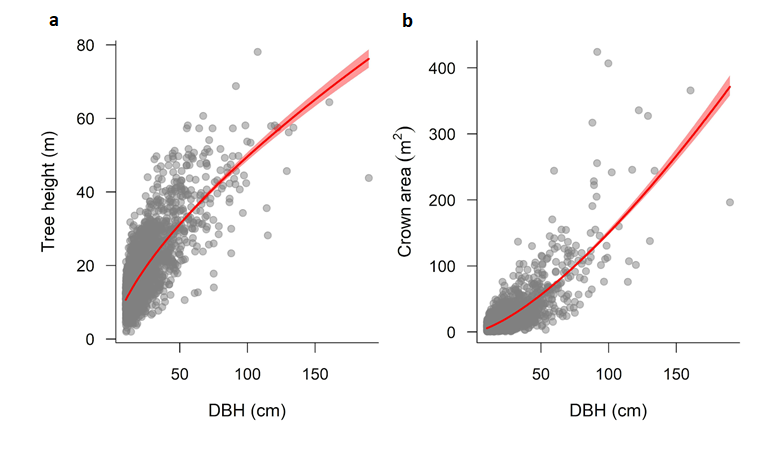
**Supplementary Fig. 8: Tree DBH as a predictor of tree height and crown area.** Diameter at breast height (DBH; cm) explaining variation in tree height (metres) and crown area (m^2^) across the SAFE landscape in Malaysian Borneo. Each point corresponds to an individual tree locally measured for a total of 3248 trees in the 1-ha Global Ecosystems Monitoring (GEM) permanent plots. We used these data to fit allometric equations for predicting the H and CA of trees that were not measured (n =10393). Fitted curves in red highlighting relationships captured by non-linear least-square models and 95% confidence intervals.

**Supplementary Methods 5. Field-estimated versus LiDAR estimated canopy heights**

Canopy height was estimated from the forest plot datasets collected in January 2013, December 2013, November 2014, December 2015 and February 2017. We calculated the crown-area weighted height of each plot, which is comparable to top-of-the-canopy height measured by LiDAR [^11,12^](https://paperpile.com/c/rfZfnr/0esZF+5zOdu), where the height of each individual tree is weighted by the horizontally projected crown area. The field-estimated canopy height agreed well with the LiDAR-based canopy height for both LiDAR surveys (Supplementary Fig. 9). Residual Standard Errors (RSE) were 8.36 m and 6.13 m for the first and second LiDAR-based TCH and field-estimated TCH relationships.

**Supplementary Fig. 9: LiDAR- and field-based forest canopy height.** Field-based top-of-canopy height (TCH) from 38 permanent plots measured in (a) November 2014 and (b) February 2017, compared with the LiDAR-estimated TCH of the same plots derived from LiDAR surveys in November 2014 and April 2016, respectively. Black dots represent the permanent plots (and missing plots are due to the presence of clouds during the first and/or second flights). The red lines represent predicted values from a non-linear regression LiDAR-based TCH = *β_0_* (field estimated TCH) *^β1^* with the shaded red area as the 95% confidence intervals. Residual Standard Errors (RSE) are reported for each model.


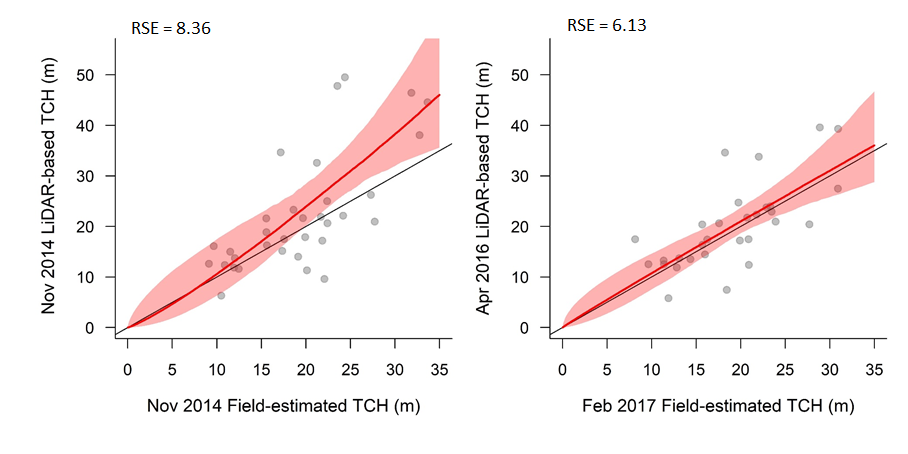


**a**

**b**

**Supplementary Methods 6. Environmental control over TCH change during the ENSO**

Change in top-of-canopy height (ΔTCH) was modelled as a function of topographic position index (TPI), the 2014 estimate of canopy height (TCH_2014_) and distance from oil palm plantations (D_edge_). We compared linear models containing these variables with the non-linear model y = a − be^(−cx)^, where a includes TPI, TCH_2014_, as well as the interaction terms TPI x TCH_2014_ and D_edge_ x TCH_2014_, and − be^(−cx)^ as an asymptotic component that represents the saturation of ΔTCH with D_edge_, denoted by x in the model. An asymptotic component in the model is more ecologically meaningful to investigate the edge effects on forest dynamics [^13^](https://paperpile.com/c/rfZfnr/jomBj). The models were fitted using the nls function in R [^14^](https://paperpile.com/c/rfZfnr/89PcR).

After comparing all the models using AIC (SI Table 1), the following was selected:

ΔTCH = *β_0_* + *β_1_* TPI + *β_2_* TCH_2014_ + *β_3_* exp^(-^ *^β4^*^Dedge )^+ ɛ (S3)

where *β_0_* to *β_4_* are the model parameters and ɛ is the normally-distributed residual error.

**Supplementary Table 1: Specification of models fitted for comparison and model selection.** Comparison of model explanatory power of linear and nonlinear models of LiDAR estimated top-of-canopy height change (∆TCH) (n = 36,655 pixels). Model explanatory power was assessed in terms of AIC and the model with the lowest value (in bold) was selected. An intercept-only model (no relationship) and linear models including topographic position index (TPI), top-of-canopy height (TCH) and distance from oil palm plantations (OP) were tested. We compare these models with non-linear models y = a − be^(−cx)^ where a includes TPI, TCH as well as TPI x TCH and TCH x OP interactions, and − be^(−cx)^ as an asymptotic relationship that corresponds to saturation of y with x.

| **Y** | **X** | **AIC** |
| --- | --- | --- |
| ΔTCH | *β_0_* | 15846.3 |
| ΔTCH | *β_0_* + *β_1_* TPI + *β_2_* TCH_2014_ | 15770.4 |
| ΔTCH | *β_0_* + *β_1_* TPI + *β_2_* TCH_2014_ + *β_3_* D_edge_ | 15739.2 |
| ΔTCH | *β_0_* + *β_1_* exp^(-^*^β^_2_* ^Dedge )^ | 15.827.4 |
| ΔTCH | *β_0_* + *β_1_* TCH_2014_ + *β_2_* exp^(-^*^β^_3_* ^Dedge )^ | 15727.7 |
| ΔTCH | *β_0_* + *β_1_* TPI + *β_2_* exp^(-^*^β^_3_* ^Dedge )^ | 15827.2 |
| **ΔTCH** | ***β_0_* + *β_1_* TPI + *β_2_* TCH_2014_ + *β_3_* exp^(-^*^β^_4_* ^Dedge )^** | **15709.0** |
| ΔTCH | *β_0_* + *β_1_* TPI + *β_2_* TCH_2014_ + *β_3_* TPI x TCH_2014_ + *β_4_* exp^(-^*^β^_5_* ^Dedge )^ | 15717.9 |
| ΔTCH | *β_0_* + *β_1_* TPI + *β_2_* TCH_2014_ + *β_3_* D_edge_ x TCH_2014_ + *β_4_* exp^(-^*^β^_5_* ^Dedge )^ | 15884.5 |

**Supplementary Methods 7. Spatial auto-correlation modelling**

Spatial autocorrelation results in underestimation of the true uncertainty in the fitted parameter values [^15^](https://paperpile.com/c/rfZfnr/txpQz). We incorporated a spatial correlation structure in the model using the nlme function in R to estimate model coefficients by maximum likelihood estimation [^16^](https://paperpile.com/c/rfZfnr/zp1V5), and spatial autocorrelation in the residual error ε was modelled using an exponential function using corExp(form = ~ X +Y), where X and Y are the plot coordinates. Although nlme is a mixed-effects modelling function, we employed it to account for spatial autocorrelation and did not include any random variables. This analysis was restricted to 5,000 randomly selected pixels from 36,655 pixels because spatial modelling using nlme is time consuming and memory demanding.

To test whether a 5,000-pixel subset is sufficient to estimate unbiased parameter values, we first ran 24 randomised permutations of the equation 2 with the spatial autocorrelation structure for randomly selected 3,000-, 4,000- and 5,000-pixel subsets from the 36,655-pixel dataset. We then generated the median and coefficient variation (CV %) of parameter values for the assessment of model stability with increasing subset sizes (Table S2). We also ran 24 randomised permutations of the equation 2 with no spatial autocorrelation structure to investigate whether median parameter values differed from parameter values when using the full dataset (Table S3). Given (i) the considerably smaller CV for the 5,000-pixel subset and (ii) the similar median parameter values of 24 5,000-pixel subsets to the full dataset’s parameters, we demonstrate the consistency of 5,000-pixel subsets to predict canopy height change across the landscape. The function intervals in R was used to predict the 95 % confidence intervals (CI_model_), and then adjusted to the full dataset (CI_corrected_) as in CI_corrected_ = CI_model_ * (n / N)^0.5^, where n is the number of variables used to estimate the parameter values in the model (5,000 pixels) and N the total number of pixels in our analysis (36,655 pixels).

In Supplementary Methods 10, we plot out the individual curves obtained by subsampling 5000 pixels at random. This figure gives a more complete impression of the uncertainty arising from variation in parameter estimates (and particularly the exponent *β_4_* ). The median predictions shown in Fig. 5 and supplementary Fig. 13 are seen to be reliable indicators of the trend in height growth with distance from edge, initial height and topographic position.

**Supplementary Table 2:** **Comparison of nonlinear mixed models with spatial autocorrelation structure with increasing number of pixels.** Median and coefficient of variation (CV) in % of parameter estimates of the model ΔTCH = *β_0_* + *β_1_* TPI + *β_2_* TCH_2014_ + *β_3_* exp ^(-^*^β^_4_* ^Dedge)^ + ɛ with spatial autocorrelation structure for 24 randomly selected permutations of 3,000- , 4,000- and 5,000-pixel subsets.

| **Parameters** | Median parameter values (Coefficient of variation %) | | |
| --- | --- | --- | --- |
|  | 3,000 pixels | 4,000 pixels | 5,000 pixels |
| *β_0_* | 0.6055 (10.7) | 0.6321 (9.9) | 0.6576 (7.5) |
| *β_1_* | -0.0204 (14.6) | -0.0218 (13.5) | -0.0214 (12.3) |
| *β_2_* | -0.0171 (25.9) | -0.0176 (17.5) | -0.0172 (21.5) |
| *β_3_* | -1.0894 (237.6) | -0.9981 (60.1) | -0.8553 (18.6) |
| *β_4_* | 0.0061 (275.2) | 0.0057 (182.2) | 0.0049 (48.5) |

**Supplementary Table 3: Comparison of 24 permutations of models with and without spatial autocorrelation structure.** Comparison of the median parameters and 95% confidence intervals (CI) of the model ΔTCH = *β_0_* + *β_1_* TPI + *β_2_* TCH_2014_ + *β_3_* exp ^(-^*^β^_4_* ^Dedge)^ + ɛ with no spatial autocorrelation structure for all pixels (36,655), for randomly selected subsets of 5,000 pixels and with the inclusion of an exponential spatial autocorrelation structure for a randomly-chosen 5,000-pixel dataset.

| **Parameters** | **Median parameters values**  **(median 95% CI)** | | |
| --- | --- | --- | --- |
|  | **No spatial autocorrelation (all 36,655 pixels)** | **No spatial autocorrelation (5,000 pixels)** | **Spatial autocorrelation**  **(5,000 pixels)** |
| *β_0_* | 0.6804  (0.6506- 0.7102) | 0.6634  (0.6300- 0.6931) | 0.6576  (0.6123- 0.7009) |
| *β_1_* | -0.0248  (-0.0261- -0.0234) | -0.02434  (-0.0257- -0.0230) | -0.02142  (-0.0233- -0.0196) |
| *β_2_* | -0.0196  (-0.0222- -0.0171) | -0.0193  (- 0.0219- -0.0168) | -0.0172  (- 0.0197- -0.0146) |
| *β_3_* | -0.9360  (-1.0202 - -0.8518) | -0.9180  (-1.0051- -0.8309) | -0.8553  (-0.9750- -0.7464) |
| *β_4_* | 0.0040  (0.0033- 0.0047) | 0.0043  (0.0035- 0.00519) | 0.0049  (0.0033- 0.0064) |

**Supplementary Methods 8. A predictive map of canopy height change across the SAFE landscape**

A predicted ΔTCH map based on equation 1 depicts the canopy change variation across the entire studied landscape with varying TCH_2014_, TPI and distance from oil palm plantations (Supplementary Fig. 10).


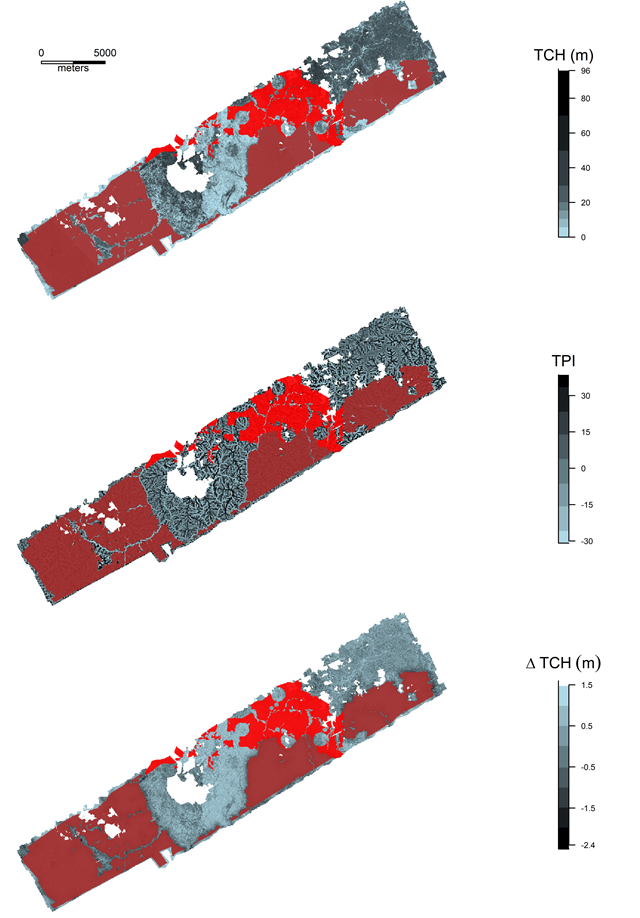


**a**

**b**

**c**

**Supplementary Fig. 10**: **Canopy height change prediction map for the Stability of Altered Forest Ecosystems (SAFE) Project landscape.** Prediction map of top-of-canopy height change (ΔTCH) change across the SAFE landscape as a function of top-of-canopy height (TCH), topographic position index (TPI) and distance from oil palm plantations according to equation 1 with shades of grey depicting the magnitude of TCH change. The brown area corresponds to oil palm plantations and the red area to salvage logging that occurred between the two LiDAR surveys. White pixels are NA data due to the presence of clouds.

**Supplementary Methods 9. Aboveground biomass (AGB) estimates**

Estimates of aboveground biomass (AGB) density were made in the permanent forest plots in Nov 2014, Dec 2015 and Feb 2017 [^17^](https://paperpile.com/c/rfZfnr/Qq2A). Aboveground wood density in these plots was estimated using the BIOMASS package in R, which draws on global databases of wood density and allometry to make its estimates, and accounts for uncertainty associated with both field measurement errors and uncertainty in allometric models [^18^](https://paperpile.com/c/rfZfnr/kkQI). We firstly estimated aboveground biomass of individual trees (in Mg) for each census using Chave and colleagues [^19^](https://paperpile.com/c/rfZfnr/4wju) pantropical biomass equation: AGB = 0.0673 x ( D x H x D^2^ ) ^0.976^. The WD was either attributed to an individual at a species, genus, family or stand level. Given data from two censuses, annual productivity was calculated as the AGB growth of all stems present in both censuses, annual mortality was the AGB lost in dead trees, and annual AGB change was the net gain all corrected for a one-year period. Field estimated annual net AGB change rates were considered constant within censuses, and AGB in April 2016 (same time as the second LiDAR survey) was estimated based on the annual AGB change rate between the Dec 2015 and Feb 2017 censuses corrected for a 4-month period.

The relationship between TCH and AGB was not strong enough to capture changes in AGB robustly and hence we opted to predict canopy height change across the landscape (Supplementary Fig. 11).

**
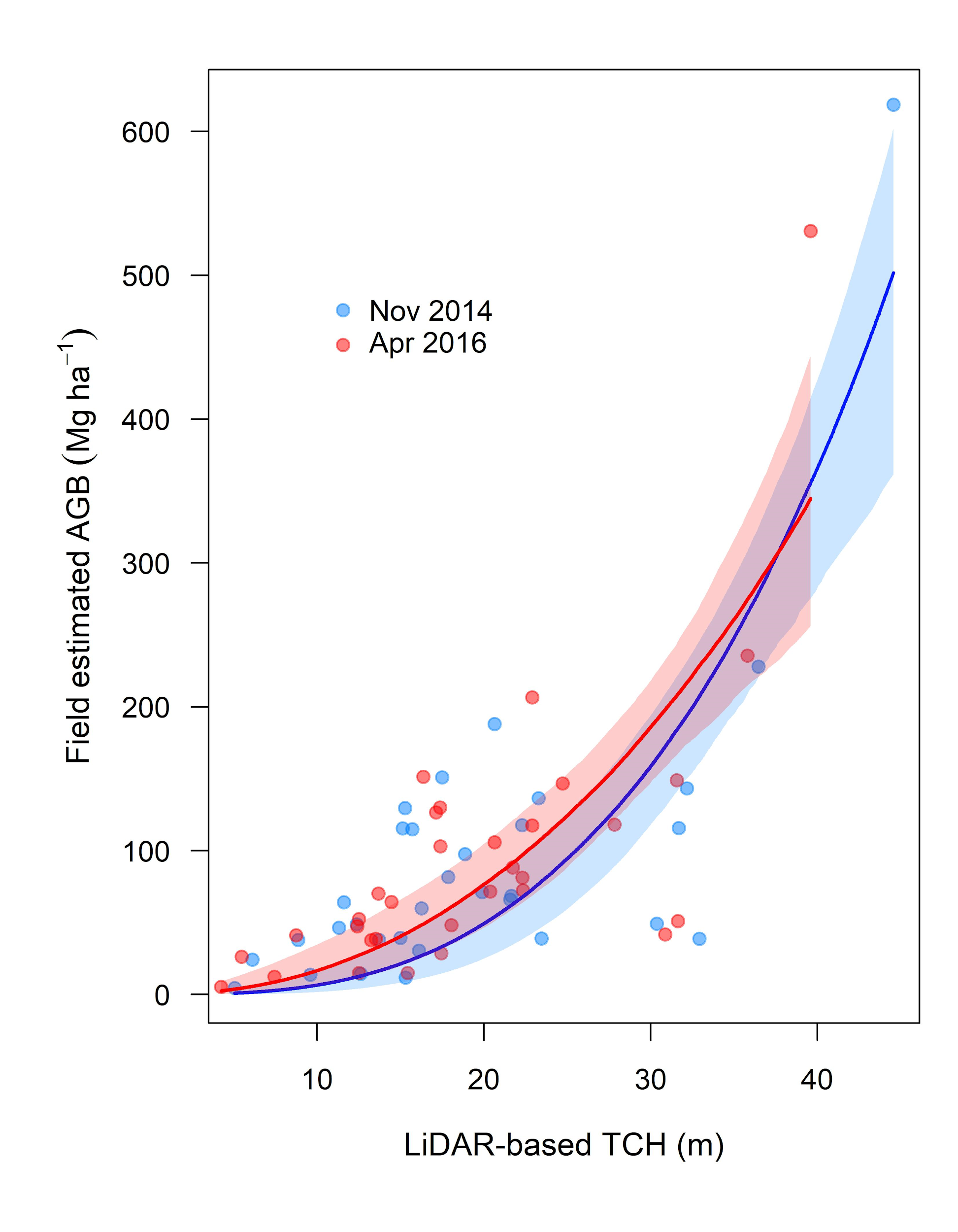
**

**Supplementary Fig. 11: Aboveground biomass versus top-of-canopy height.** LiDAR-based top-of-canopy height (TCH) (m) versus field-estimated aboveground biomass (AGB; Mg ha^-1^) from 38 permanent plots in between November 2014 (blue) and April 2016 (red). Black dots represent the permanent plots (and missing plots are due to the presence of clouds during the first and/or second flights). The red and blue lines represent predicted values from a non-linear regression field estimated AGB = *β_0_* (LiDAR-based TCH) *^β^_1_* with shaded areas as the 95% confidence intervals.

We also used the field data to predict the influence of field estimated aboveground biomass (AGB) change on LiDAR-based canopy height change. Field estimated AGB change had a correlation of 0.77 with TCH change (P < 0.001; Supplementary Fig. 12). We demonstrated that a reduction in TCH was the result of a decline in AGB change. The two components of AGB change are the biomass of dead trees (AGB mortality) and the biomass accumulation of surviving trees (AGB growth). During the ENSO, AGB growth accounted for 70% of the effects on net AGB change (P < 0.001), whilst mortality of dead trees accounted for 25% of the total AGB change (P < 0.001). No significant interaction between AGB mortality and AGB growth was found.


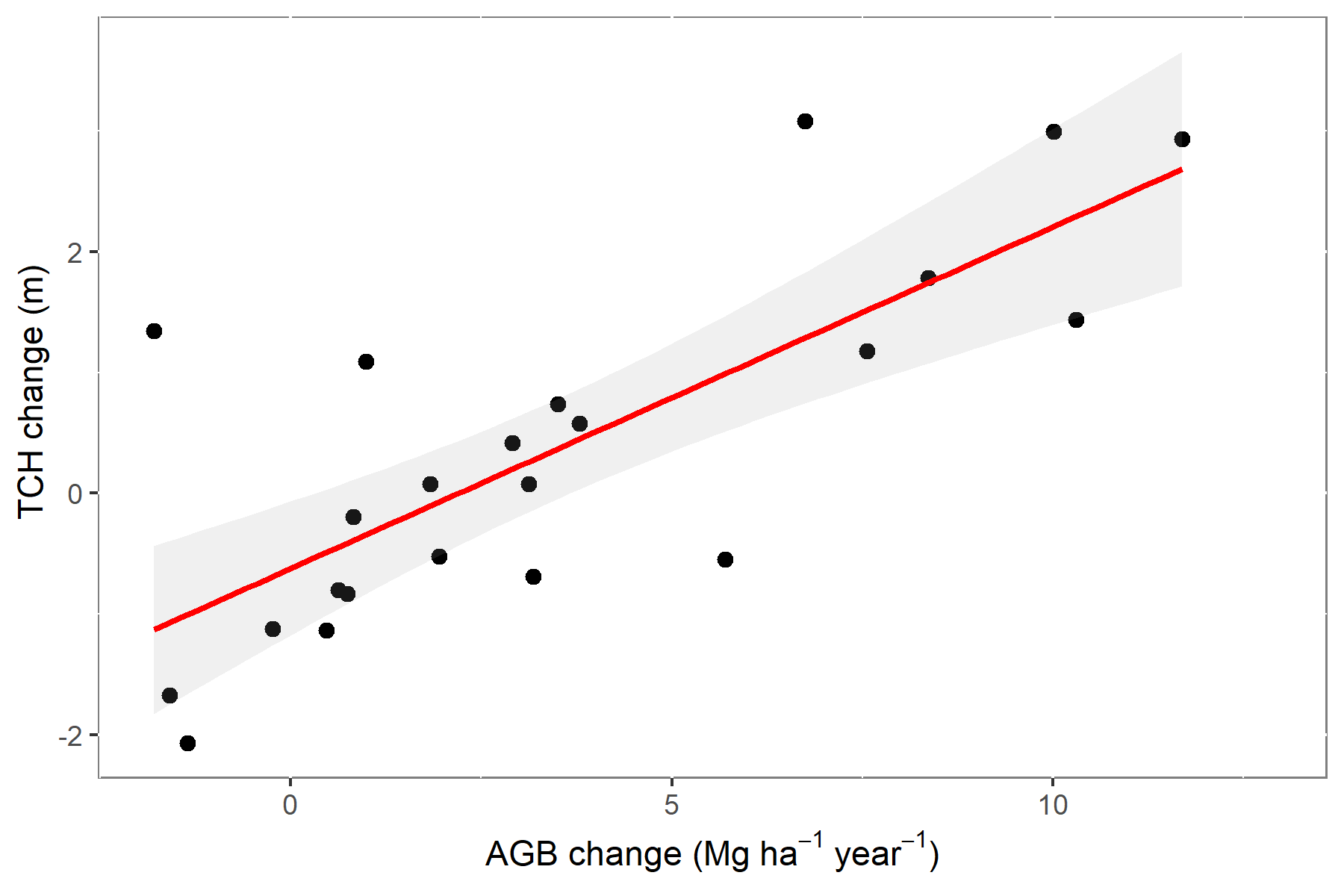


**Supplementary Fig. 12: Aboveground biomass change versus top-of-canopy height change.** Relationship between LiDAR-based top-of-canopy height (TCH) change (m) versus field estimated aboveground biomass (AGB) change ( Mg ha^-1^). 38 permanent plots with tree measurements across a human-modified landscape with tree measurements made in November 2014, December 2015 and February 2017. Each dot represents one permanent plot (missing plots are due to the presence of clouds) and the red lines represent predicted values from a linear regression TCH change = *β_0_* (AGB change) *^β^_1_* with the shaded red area as the 95% CI. Field estimated net AGB change was estimated for each census interval as the difference between standing biomass at the end and the beginning of the interval divided by the census length. Field estimated net AGB change rates were considered constant within censuses thereby AGB in April 2016 was then estimated based on the annual AGB change rate between the Dec 2015 and Feb 2017 censuses corrected for a 4-month period.

**Supplementary Methods 10. Predicted effects of fragmentation and topography on canopy height change**


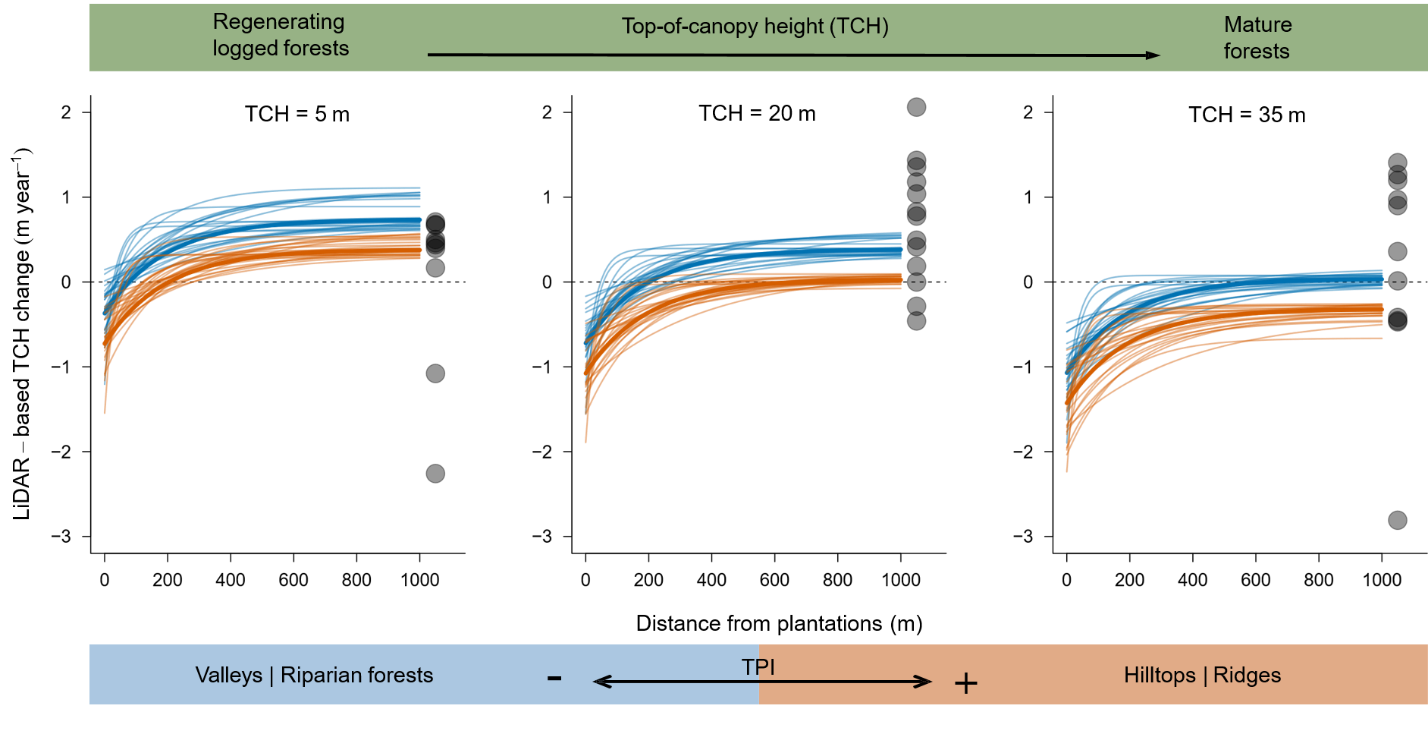


**Supplementary Fig. 13: Predicted fragmentation and topographic effects on canopy height change from 24 subsets of the dataset.** Predicted effects of distance from oil palm plantations and topographic position (TPI) on the canopy height growth (i.e. TCH change, in metres per year) of forests at different successional stages, recorded by airborne LiDAR surveys made at the beginning and end of the 2015-2016 El Niño Southern Oscillation (ENSO) event in Malaysian Borneo. Canopy height growth was predicted by fitting nonlinear models (with a spatial autocorrelation structure) to 24 subsets of the datasets, each composed of 5000 measurements. Predictions from the 24 models are plotted as fine lines, and predictions based on median parameter values are shown in as thick lines. Predictions as shown for short, medium and tall canopies with initial heights of 5 m, 20 m and 35 m. Valley-bottom (blue) and hilltop (orange) curves were predicted using TPI values of -8.2 and 9.0, corresponding to the 5th and 95th quantiles of TPI. The translucent black solid points on the right side of each panel correspond to the pre-ENSO canopy height growth estimates between Jan 2013 and November 2014, obtained from 38 permanent forest plots. This figure gives a more complete picture of the uncertainty between model runs to that shown in Fig. 5 of the main text, where variation across models was summarised into a single line of best fit and associated confidence intervals.

**REFERENCES**

1. [Asner, G. P. et al. Carnegie Airborne Observatory-2: Increasing science data dimensionality via high-fidelity multi-sensor fusion. Remote Sens. Environ. **124**, 454–465 (2012).](http://paperpile.com/b/rfZfnr/wtQ9u)

2. [Roussel, J.-R., Caspersen, J., Béland, M., Thomas, S. & Achim, A. Removing bias from LiDAR-based estimates of canopy height: Accounting for the effects of pulse density and footprint size. Remote Sens. Environ. **198**, 1–16 (2017).](http://paperpile.com/b/rfZfnr/WU2Cl)

3. [Khosravipour, A., Skidmore, A. K., Isenburg, M., Wang, T. & Hussin, Y. A. Generating Pit-free Canopy Height Models from Airborne Lidar. Photogrammetric Engineering & Remote Sensing **80**, 863–872 (2014).](http://paperpile.com/b/rfZfnr/Fryr)

4. [Asner, G. P. & Mascaro, J. Mapping tropical forest carbon: Calibrating plot estimates to a simple LiDAR metric. Remote Sens. Environ. **140**, 614–624 (2014).](http://paperpile.com/b/rfZfnr/PXen)

5. [Asner, G. P. et al. Mapped aboveground carbon stocks to advance forest conservation and recovery in Malaysian Borneo. Biol. Conserv. **217**, 289–310 (2018).](http://paperpile.com/b/rfZfnr/PVBQV)

6. [Gobakken, T. G. & Næsset, E. N. Assessing effects of laser point density, ground sampling intensity, and field sample plot size on biophysical stand properties derived from airborne laser scanner data. Can. J. For. Res. (2008) doi:](http://paperpile.com/b/rfZfnr/3sgz)[10.1139/X07-219](http://dx.doi.org/10.1139/X07-219)[.](http://paperpile.com/b/rfZfnr/3sgz)

7. [Metcalfe, P., Beven, K. & Freer, J. Dynamic TOPMODEL: A new implementation in R and its sensitivity to time and space steps. Environmental Modelling & Software **72**, 155–172 (2015).](http://paperpile.com/b/rfZfnr/veep)

8. [Muscarella, R., Kolyaie, S., Morton, D. C., Zimmerman, J. K. & Uriarte, M. Effects of topography on tropical forest structure depend on climate context. J. Ecol. **108**, 145–159 (2020).](http://paperpile.com/b/rfZfnr/gjk2)

9. [H’edl, R. et al. A new technique for inventory of permanent plots in tropical forests: a case study from lowland dipterocarp forest in Kuala Belalong, Brunei Darussalam. Blumea - Biodiversity, Evolution and Biogeography of Plants **54**, 124–130 (2009).](http://paperpile.com/b/rfZfnr/uyYxr)

10. [Pfeifer, M. et al. Mapping the structure of Borneo’s tropical forests across a degradation gradient. Remote Sens. Environ. **176**, 84–97 (2016).](http://paperpile.com/b/rfZfnr/gnGYz)

11. [Kent, R., Lindsell, J. A., Laurin, G. V., Valentini, R. & Coomes, D. A. Airborne LiDAR Detects Selectively Logged Tropical Forest Even in an Advanced Stage of Recovery. Remote Sensing **7**, 8348–8367 (2015).](http://paperpile.com/b/rfZfnr/0esZF)

12. [Pang, Y., Lefsky, M., Andersen, H.-E., Miller, M. E. & Sherrill, K. Validation of the ICEsat vegetation product using crown-area-weighted mean height derived using crown delineation with discrete return lidar data. Can. J. Remote Sens. **34**, S471–S484 (2008).](http://paperpile.com/b/rfZfnr/5zOdu)

13. [Qie, L. et al. Long-term carbon sink in Borneo’s forests halted by drought and vulnerable to edge effects. Nature Communications vol. 8 (2017).](http://paperpile.com/b/rfZfnr/jomBj)

14. [Team, R. C. R: ALanguage and Environment for Statistical Computing. Foundation for Statistical Computing, Vienna, Austria. 2017. Available online: www. r-project. org (accessed on 14 Febuary 2019) (2018).](http://paperpile.com/b/rfZfnr/89PcR)

15. [Ploton, P. et al. Spatial validation reveals poor predictive performance of large-scale ecological mapping models. Nat. Commun. **11**, 4540 (2020).](http://paperpile.com/b/rfZfnr/txpQz)

16. [Wedeux, B. et al. Dynamics of a human-modified tropical peat swamp forest revealed by repeat lidar surveys. Glob. Chang. Biol. (2020) doi:](http://paperpile.com/b/rfZfnr/zp1V5)[10.1111/gcb.15108](http://dx.doi.org/10.1111/gcb.15108)[.](http://paperpile.com/b/rfZfnr/zp1V5)

17. [Jucker, T. et al. Estimating aboveground carbon density and its uncertainty in Borneo’s structurally complex tropical forests using airborne laser scanning. Biogeosciences (2018).](http://paperpile.com/b/rfZfnr/Qq2A)

18. [Réjou‐Méchain, M., Tanguy, A., Piponiot, C., Chave, J. & Hérault, B. biomass : an r package for estimating above‐ground biomass and its uncertainty in tropical forests. Methods Ecol. Evol. **8**, 1163–1167 (2017).](http://paperpile.com/b/rfZfnr/kkQI)

19. [Chave, J. et al. Improved allometric models to estimate the aboveground biomass of tropical trees. Glob. Chang. Biol. **20**, 3177–3190 (2014).](http://paperpile.com/b/rfZfnr/4wju)
