# Supplementary material for: Recovery of logged forest fragments in a human-modified tropical landscape during the 2015-16 El Niño
Source: Nat Commun. 2021 Mar 9;12:1526. doi: 10.1038/s41467-020-20811-y (PMC7943823; doi:10.1038/s41467-020-20811-y)
Supplement: Supplementary file 2 — Reporting Summary [file 41467_2020_20811_MOESM2_ESM.pdf]

nature research

Corresponding author(s): David Coomes
Matheus Nunes

Last updated by author(s): 19/10/2020

Reporting Summary

Nature Research wishes to improve the reproducibility of the work that we publish. This form provides structure for consistency and transparency in reporting. For further information on Nature Research policies, see our Editorial Policies and the Editorial Policy Checklist.

Statistics

For all statistical analyses, confirm that the following items are present in the figure legend, table legend, main text, or Methods section.

n/a	Confirmed	
☐	☒	The exact sample size (*n*) for each experimental group/condition, given as a discrete number and unit of measurement
☐	☒	A statement on whether measurements were taken from distinct samples or whether the same sample was measured repeatedly
☐	☒	The statistical test(s) used AND whether they are one- or two-sided<br>*Only common tests should be described solely by name; describe more complex techniques in the Methods section.*
☐	☒	A description of all covariates tested
☒	☐	A description of any assumptions or corrections, such as tests of normality and adjustment for multiple comparisons
☐	☒	A full description of the statistical parameters including central tendency (e.g. means) or other basic estimates (e.g. regression coefficient) AND variation (e.g. standard deviation) or associated estimates of uncertainty (e.g. confidence intervals)
☐	☒	For null hypothesis testing, the test statistic (e.g. *F*, *t*, *r*) with confidence intervals, effect sizes, degrees of freedom and *P* value noted<br>*Give P values as exact values whenever suitable.*
☒	☐	For Bayesian analysis, information on the choice of priors and Markov chain Monte Carlo settings
☒	☐	For hierarchical and complex designs, identification of the appropriate level for tests and full reporting of outcomes
☒	☐	Estimates of effect sizes (e.g. Cohen's *d*, Pearson's *r*), indicating how they were calculated

Our web collection on statistics for biologists contains articles on many of the points above.

Software and code

Policy information about availability of computer code

Data collection	Crown project of trees collected using Field-Map technology (IFER, Ltd., Jílové u Prahy, Czech Republic)<br>Tree height measurement: Impulse 200 laser rangefinder (Laser Technology Inc., Colorado, USA)<br>Pléiades satellite constellation imagery was used to classify land use using QGIS 3.2.3<br>Hemispherical photographs were taken using Sigma 4.5 mm f/2.8 EX DC HSM circular fisheye lens. The photos were thresholded in ImageJ (version 1.51j8) using Auto Threshold and IsoData. The photos were analysed with Hemisfer software version (3.0).
Data analysis	Triangulated irregular network algorithm: LASTools (rapidlasso, GmbH; Gilching, Germany)<br>CHM calculation: LASTools (rapidlasso, GmbH; Gilching, Germany)<br>Calculation of distance of forests from oil palm plantations: R software (2018)<br>Calculation of TPI from digital elevation model: R software (2018)<br>Calculation of TWI from digital elevation model: R software (2018)<br>Software R Core Team (2018) was used to analyse the data and develop all linear multiple models and a nonlinear mixed effects model.

For manuscripts utilizing custom algorithms or software that are central to the research but not yet described in published literature, software must be made available to editors and reviewers. We strongly encourage code deposition in a community repository (e.g. GitHub). See the Nature Research guidelines for submitting code & software for further information.

Data

Policy information about availability of data

All manuscripts must include a data availability statement. This statement should provide the following information, where applicable:

- Accession codes, unique identifiers, or web links for publicly available datasets
- A list of figures that have associated raw data
- A description of any restrictions on data availability

Repeated canopy height data, topographic position index and distance of forests from oil palm plantations generated from repeat LiDAR surveys and analysed during the study have been deposited in the UK Centre for Ecology and Hydrology and made publicly available with the identifier https://doi.org/10.5285/534838c8-0e1f-4a04-a837-2e19a4e93797. Microclimate data across permanent plots are openly available online from http://doi.org/10.5281/zenodo.1441585.

Field-specific reporting

Please select the one below that is the best fit for your research. If you are not sure, read the appropriate sections before making your selection.

☐ Life sciences ☐ Behavioural & social sciences ☒ Ecological, evolutionary & environmental sciences

For a reference copy of the document with all sections, see nature.com/documents/nr-reporting-summary-flat.pdf

Ecological, evolutionary & environmental sciences study design

All studies must disclose on these points even when the disclosure is negative.

Study description	Repeat high-density airborne LiDAR data across 3,300 ha in Malaysian Borneo (N = 36,655 pixels) were used to investigate forest canopy growth. Field data with repeat tree and canopy measurements from 38 small permanent plots (25 x 25 m in size) and 8 large permanent plots (1 ha) were used to elucidate the mechanisms driving changes in canopy height.<br>Microclimate data collection resulted in a total of 939,388 coupled T and RH readings.
Research sample	Repeat airborne laser scanning surveys covered the world's largest forest fragmentation experiment, the Stability of Altered Forest Ecosystems (SAFE) Project in Sabah, Malaysian Borneo. Plot networks with tree and canopy measurements repesent forests at different successional stages. The entire SAFE Project experimental site has an area of 7200 ha. The experimental site currently connects a Virgin Jungle Reserve (VJR) of 2200 ha to a large area of forest (greater than 1 million ha). Most of the large expanse has been through either one or two rotations of selective logging. The forest modification gradient reproduces the real-world pattern of habitat conversion in Borneo, ensuring that phenomena observed in the study should be directly pertinent to policy issues in the region. All regenerating logged forests covered by repeat LiDAR during the El Niño were selected as the sample choice thus only regrowth of natural forests was evaluated.
Sampling strategy	Airborne laser scanning surveys covered an area of 3,300 ha (N = 36,655 pixels). However, spatial analysis was restricted to 5,000 randomly selected pixels because spatial modelling using nlme is time consuming and memory demanding. To test whether a 5,000-pixel subset is sufficient to estimate unbiased parameter values, we first ran 24 randomised permutations of the equation 2 with the spatial autocorrelation structure for randomly selected 3,000-, 4,000- and 5,000-pixel subsets from the 36,679-pixel dataset. We then generated the mean and coefficient variation (CV %) of parameter values for the assessment of model stability with increasing subset sizes (Table S2). We also ran 24 randomised permutations of the equation 2 with no spatial autocorrelation structure to investigate whether mean parameter values differed from parameter values when using the full dataset (Table S3). Given (i) the considerably smaller CV for the 5,000-pixel subset and (ii) the similar mean parameter values of 24 5,000-pixel subsets to the full dataset's parameters, we demonstrate the consistency of 5,000-pixel subsets to predict canopy height change across the landscape.<br><br>The SAFE permanent plot network with microclimate, tree and canopy openness measurements and its sampling design have been described in detail in Ewers et al., (2011) <https://royalsocietypublishing.org/doi/full/10.1098/rstb.2011.0049>. In summary, the SAFE Project represents an advance on existing experiments in that it: (i) allows discrimination of the effects of landscape-level forest cover from patch-level processes; (ii) is designed to facilitate the unification of a wide range of data types on ecological patterns and processes that operate over a wide range of spatial scales; (iii) has greater replication than existing experiments; (iv) incorporates an experimental manipulation of riparian corridors; and (v) embeds the experimentally fragmented landscape within a wider gradient of land-use intensity than do existing projects.<br>The Global Ecosystem Monitoring (GEM) plot network with LAI, branch fall and leaflitter measurements is within a disturbance gradient from heavily to moderately logged forest sites and old-growth forests. All plots had a planimetric area of 1 ha, divided into 25 subplots of 20 m × 20 m on reasonably homogenous soil parent material and soil type. A plot size of 1 ha (planimetric) is commonly chosen because it is greater than the scale of typical tree fall events. GEM plots website can be found on <http://gem.tropicalforests.ox.ac.uk/page/resources>.<br>Both SAFE and GEM plot networks use the RAINFOR protocol for measurements.
Data collection	The first LiDAR data were acquired in November 2014 using a Leica ALS50-II LiDAR sensor flown by NERC's Airborne Research Facility. The second LiDAR survey was conducted by the ASU Global Airborne Observatory (GAO; formerly the Carnegie Airborne Observatory 71) in April 2016.<br>Satellite imagery was used to classify land use, and calculate distance from forest to oil palm edge. Earth Imaging data from the Pléiades satellite constellation (EADS Astrium), acquired over the SAFE landscape in June 2016, were classified visually to define boundaries between forest and plantations.<br>Canopy height loss recorded by LiDAR could be a response to leaf loss, branch loss or tree death, while height gain could arise from

leaf gain or upward stem growth. We used canopy height, Plant Area Index (PAI), branch fall and leaf litter from two separate permanent plot networks within the SAFE landscape to investigate how the 2015-16 ENSO event affected the canopy and which factors were driving the changes observed by LiDAR. More specifically, we used tree and canopy openness measurements from 38 permanent forest inventory plots (SAFE plots established by the co-authors of the study Martin Svátek, Jakub Kvasnica, Martin Rejžek, Radim Matula) established in 2011 (each 25 x 25 m in size), as well as PAI, branch fall and leaf litter measurements from 8 1-ha Global Ecosystems Monitoring (GEM) plots established by the co-author of this study Terhi Riutta. Although these estimates cannot be used to directly validate the LiDAR measurements, they help us investigate the likely mechanisms driving canopy changes during the ENSO event.
The continuous microclimate data from a plot network is publicly available on <https://zenodo.org/communities/safe/?page=1&size=20>.
Daily precipitation was systematically recorded from June 2013 to October 2018 at a weather station at the SAFE field station.

Timing and spatial scale	Repeat LiDAR surveys were conducted across a 3,300 ha forest landscape in Sabah, Malaysian Borneo, during the 2015-16 El Niño that ocurred in the region between January 2015 and April 2016. In order to investigate the effects of extreme climatic events on the canopy of regenerating forests, we conducted a second LiDAR survey in the end of the El Niño. The first LiDAR data were acquired in November 2014. The second LiDAR survey was conducted in April 2016.<br>Trees were measured in the field in January 2013, December 2013, November 2014, December 2015 and February 2017 from 38 plots of 25 x 25 m and 8 1-ha plots.<br>Canopy openness measurements were made in the SAFE plots between November 2014 and February 2017 from 38 plots of 25 x 25 m .<br>PAI data from the 8 1-ha GEM plots were continuously measured between August 2013 and June 2018 to create a PAI time-series.<br>We measured branch fall (July 2014 and - July 2017) and litterfall (January 2013 - June 2018) from the 8 1-ha GEM plots.<br>Daily precipitation was systematically recorded from June 2013 to October 2018.<br>Microclimate data were collected between May 2013 and August 2017 from 113 plots, resulting in a total of 939,388 coupled T and RH readings.
Data exclusions	The total area with repeated LiDAR flights covered 24,120 ha of forest and oil palm plantation mosaic. To derive regenerating logged forest pixels from the dataset, we excluded 9587 ha of oil palm plantations and 2,500 ha of forest that was clear-cut ("salvage logged") between the two LiDAR surveys. Loss of biomass with logging can be due to the immediate damage caused by felling the selected trees, incidental damage to surrounding trees caused by the felled trees, and the infrastructure built for removing the logs out of the forest 75. To avoid potential effects of logging that occurred in the interim of both flights on the surrounding forests due to infrastructure, pixels within 200 m of the clear-cut areas were also removed. Roads and their adjacent areas within 30 m were also removed due to their intrinsic differences in land cover compared to forest canopies. No other land-use types remained within the resulting study area. Finally, since LiDAR estimates can be affected by point density, biases arising from differences in point density were removed from the dataset. We demonstrate that an underestimation of tree height associated with point density < 10 points m-2 in the NERC dataset may have contributed to an overestimation of TCH change (Supplementary Fig. 2) and, therefore, we removed these pixels (~ 62% of the dataset). We also assessed the influence of point density variation in the GAO data and did not find any influence of point density of TCH change estimation (Supplementary Fig. 3, 4). A small number of outliers that may have resulted from anomalies in the processing of the DTM and TCH or small misalignments were still detected, and thus we trimmed the lower and upper 1% of all TCH change values with the intention to eliminate unrealistic values. The final area analysed was 3301 ha.
Reproducibility	All attempts to repeat the measurement were successful. We ran 24 randomised permutations of the equation 2 to investigate whether all parameters were significant.
Randomization	It is not relevant to our study - we did not allocate any samples into groups.
Blinding	Blinding is not relevant to our study. No persons were used in the study.

Did the study involve field work? ☒ Yes ☐ No

Field work, collection and transport

Field conditions	Continuous tree and canopy measurements were done between 2013 and 2017. Field conditions included a large range of precipitation and temperatures given that field word was conducted throughout the entire period.
Location	The study is located in Sabah, Malaysian Borneo, within a region dominated by logged forests and oil palm plantations (4 38' N to 4 46' N, 116 57' to 117 42' E).
Access & import/export	Permission to conduct research in the area was granted by Sabah Biodiversity Council. We did not import or export any material.
Disturbance	No disturbance was caused by this study.

Reporting for specific materials, systems and methods

We require information from authors about some types of materials, experimental systems and methods used in many studies. Here, indicate whether each material, system or method listed is relevant to your study. If you are not sure if a list item applies to your research, read the appropriate section before selecting a response.

Materials & experimental systems

n/a	Involved in the study
☒	☐ Antibodies
☒	☐ Eukaryotic cell lines
☒	☐ Palaeontology and archaeology
☒	☐ Animals and other organisms
☒	☐ Human research participants
☒	☐ Clinical data
☒	☐ Dual use research of concern

Methods

n/a	Involved in the study
☒	☐ ChIP-seq
☒	☐ Flow cytometry
☒	☐ MRI-based neuroimaging
